# Cellobiohydrolase 1 from *Trichoderma reesei* degrades cellulose in single cellobiose steps

**DOI:** 10.1038/ncomms10149

**Published:** 2015-12-10

**Authors:** Sonia K. Brady, Sarangapani Sreelatha, Yinnian Feng, Shishir P. S. Chundawat, Matthew J Lang

**Affiliations:** 1 Department of Chemical and Biomolecular Engineering, Vanderbilt University, PMB 351604, 2301 Vanderbilt Place, Nashville, Tennessee 37235-1604, USA; 2 SMART- BioSystems and Micromechanics, National University of Singapore, 1 Create Way, #04-13/14 Enterprise Wing and #B-101, Singapore 138602, Singapore; 3 Department of Chemical and Biochemical Engineering, Rutgers, State University of New Jersey, 98 Brett Road, Room C-150A, Piscataway, New Jersey 08854, USA; 4 Department of Molecular Physiology and Biophysics, Vanderbilt University, PMB 351604, 2301 Vanderbilt Place, Nashville, Tennessee 37235-1604, USA

## Abstract

Cellobiohydrolase 1 from *Trichoderma reesei* (*Tr*Cel7A) processively hydrolyses cellulose into cellobiose. Although enzymatic techniques have been established as promising tools in biofuel production, a clear understanding of the motor's mechanistic action has yet to be revealed. Here, we develop an optical tweezers-based single-molecule (SM) motility assay for precision tracking of *Tr*Cel7A. Direct observation of motility during degradation reveals processive runs and distinct steps on the scale of 1 nm. Our studies suggest *Tr*Cel7A is not mechanically limited, can work against 20 pN loads and speeds up when assisted. Temperature-dependent kinetic studies establish the energy requirements for the fundamental stepping cycle, which likely includes energy from glycosidic bonds and other sources. Through SM measurements of isolated *Tr*Cel7A domains, we determine that the catalytic domain alone is sufficient for processive motion, providing insight into *Tr*Cel7A's molecular motility mechanism.

## Abstract (web summary)

Cellobiohydrolases are promising tools in biofuel production by hydrolysing cellulose into cellobiose. Here the authors use optical tweezers to show that Cellobiohydrolase 1 from *Tricodermia reesei* functions processively against moderate load, and likely uses multiple energy sources to fuel each step along the cellulose fibre.

## Graphical abstract

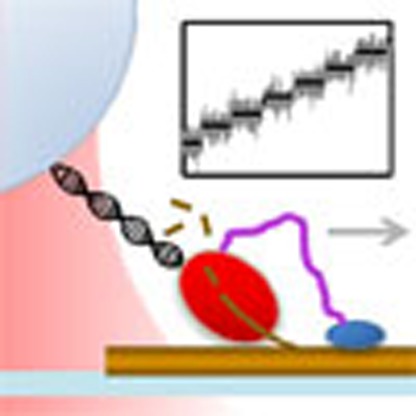

Cellulose, the most abundant polymer on the Earth^1^,^2^, is highly resistant to hydrolysis, and is degraded by a number of enzymes referred to as cellulases. Cellulases are used in many industries, including food processing, pulp and paper, and most recently, the biofuel industry, as a feedstock-derived sugar source for conversion to ethanol. Cellulose is also a major component of biofilm mats such as those in aquatic environments, pipe fouling and dental plaques^3^,^4^,^5^. Despite the remarkably diverse uses of cellulose-based products, its structural stability often leads to disposal as waste in biofuel production processes and problems due to its role in biofilm and bacterial mat stability.

The decomposition of cellulose into basic sugar components, cellobiose and glucose, is a bottleneck in cellulosic biofuel production^6^,^7^. The most common and effective industrial cellulose degradation processes include heat, mechanical and acid treatment. However, enzymatic degradation has become an attractive alternative because of its more environmentally benign nature^8^,^9^. Enzymatic processing allows for lower operating temperatures, leading to greater net energy production, milder processing conditions and minimized wear on processing units. Unfortunately, enzymes are expensive and slow. A better understanding of cellulase mechanisms could lead to decreased enzyme costs and improved economics of industrial production plants.

Enzymatic cellulose degradation occurs naturally through a system of cellulases such as those secreted by the fungus *Trichoderma reesei*. Here a mixture of cellulases serve specialized roles in cellulose and oligosaccharide hydrolysis. Cellobiohydrolase 1 from *Trichoderma reesei* (*Tr*Cel7A), representing 60% of the enzyme cocktail population, is the primary exocellulase and degrades cellulose into cellobiose^10^. Exocellulases act on crystalline regions of cellulose fibres, tend to be processive, are directionally dependent, and are thought to be powered, in part, by the energy from hydrolysis of the glycosidic bond^11^. *Tr*Cel7A has three major parts: a small carbohydrate-binding module (CBM), a larger catalytic domain (CD) and a short, 27 aa linker domain (LD) connecting the two (Fig. 1a).

Prior work using high speed atomic force microscopy (HS-AFM) tracked the motility of low concentrations of *Tr*Cel7A motors on highly crystalline (>80%) *Cladophora*-derived cellulose^12^ showing *Tr*Cel7A translocation with an average apparent velocity of 5.3±4.9 nm s^−1^ at 25 °C (refs 7, 13). Records from Igarashi *et al*.^7^ showed global pause and run states spanning up to 70 nm and observed a ‘traffic jam' tendency of cellulases to bunch up along the track. Single-molecule (SM) fluorescence studies revealed unloaded on and off rates, observing non-productive dwells as well as longer associations^13^,^14^,^15^.

Here, we designed a SM motility assay based on optical tweezers (Fig. 1d) for precision tracking of individual wild-type *Tr*Cel7A (wt*Tr*Cel7A) and isolated CD (Fig. 1b) on, primarily, filter paper-derived cellulose (∼68% crystalline)^16^, with nanometre resolution under load. Studies reveal translocation in single cellobiose steps with velocity and stepping behaviour almost identical for both constructs, indicating that the CD is independently responsible for translocation (and hydrolysis). Binding studies of isolated CBM (Fig. 1c) reveal that the presence of CBM may sometimes even hinder translocation, a small price to pay given the decreased binding of the motor when the CBM is removed, as noted in our activity studies. Additional experiments probing *Tr*Cel7a motility at elevated temperatures also provide insight into the energetic barriers of the motility cycle.

## Results

### Optical trapping assay overview

**Figure 1:**
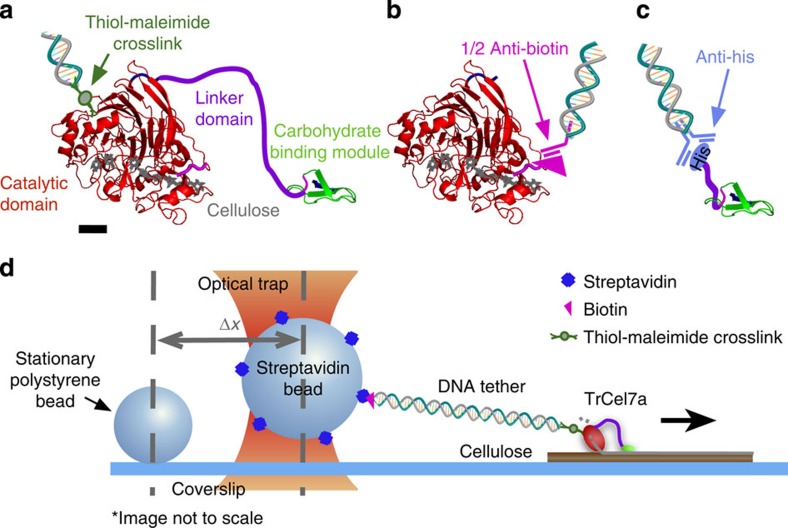
Constructs and assay schematic. Construct details and optical trap assay schematic for (**a**) wt*Tr*Cel7A, where a DNA-bound sulfo-SMCC crosslinks through available surface lysines (scale bar, 1 nm), (**b**) isolated biotin-labelled CD ligated to DNA through a ½ anti-biotin antibody and (**c**) isolated CBM tethered through a DNA-bound anti-His antibody. Structures in **a**–**c** are from PDB 7CEL and 2CBH. (**d**) A schematic of the wt*Tr*Cel7A motility assay tracks motility through a 1,010-bp tether attached to a 1.25-μm streptavidin bead held in an optical trap. Stationary fiducial beads serve to compensate for drift.

Our primary motility assay consists of full motors, wt*Tr*Cel7A, that are purified from a mixture of *T. reesei* cellulases (Sigma) using ion-exchange chromatography^17^. Motors are biotinylated, via a 1,010-bp DNA tether, and attached to a 1.25-μm streptavidin-coated polystyrene bead. Beads were trapped and placed on cellulose fibres, derived from filter paper, that are affixed to a cover glass surface. Smaller 0.75 μm beads, serving as fiducial markers for position to compensate for drift, were also affixed to the cover glass (Fig. 1d).

The validity of our drift adjustment method and 1 nm step resolution were confirmed in control experiments (Supplementary Note 2 and Supplementary Fig. 5).

### Motility characterization of wt*Tr*Cel7a

**Figure 2:**
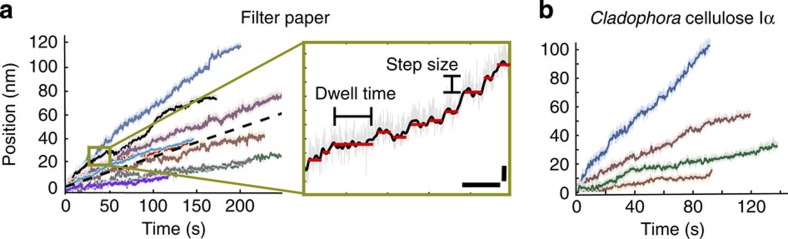
Motility traces and stepping definitions. (**a**) Sample wt*Tr*Cel7A motility traces on filter paper exhibit a range of velocities with an average of 0.25 nm s^−1^±0.16 (s.d.) dashed line. The average is representative of 180 traces from 64 separate enzymes at 21 °C. The inset reveals an enlarged region of one trace highlighting the fine stepping motion of wt*Tr*Cel7A. Scale bars are 5 s and 2 nm, respectively. (**b**) Sample *Tr*Cel7a motility traces on *Cladophora*-derived cellulose. The average velocity is 0.25 nm s^−1^±0.35 (s.d.) and is representative of 68 traces from 17 separate enzymes at 21 °C.

**Figure 3:**
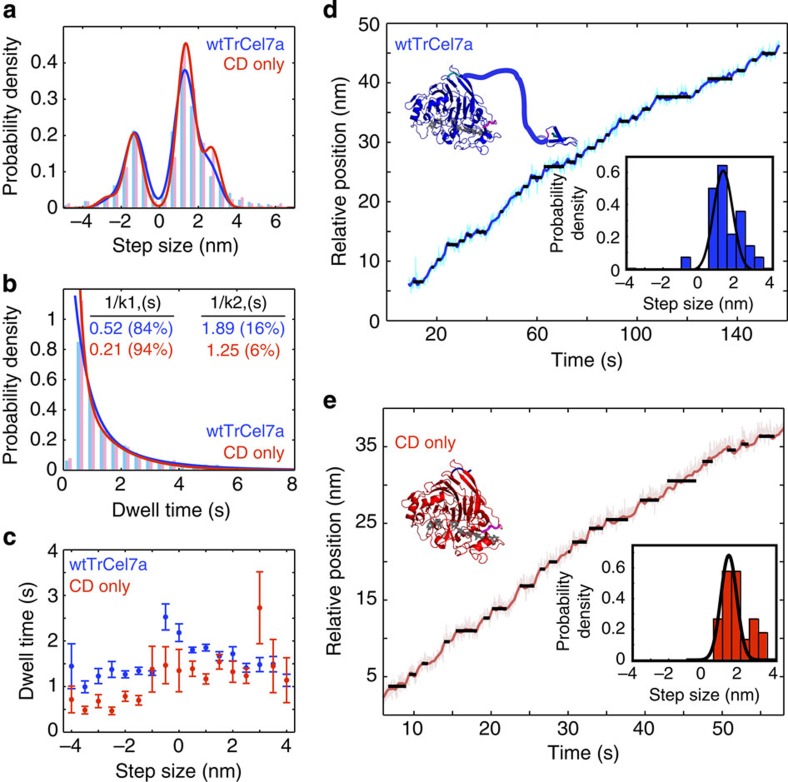
Stepping analysis of wt*Tr*Cel7A (blue) and isolated CD (red). (**a**) Step size distributions fit to Gaussian curves based on the fundamental and 2 × fundamental steps (wt*Tr*Cel7A: 1.28±0.7 nm (s.d.), *N*=1614; isolated CD: 1.34±0.6 nm (s.d.), *N*=360). (**b**) Dwell distributions fit to a double exponential with exponential time constants, 1/*k*_1_ and 1/*k*_2_, indicated in the figure. The mean dwell time of wt*Tr*Cel7A (*N*=1628) and isolated CD (*N*=369) are 1.6 and 1.2 s, respectively. (**c**) The relationship between step size and dwell time shows generally shorter dwell times associated with negative steps and isolated CD measurements (red). Error bars denote s.e.m. (**d**) and (**e**) provide sample traces with individual trace step distributions (insets) with similar behaviour between the wt*Tr*Cel7A and isolated CD constructs.

Motility of wt*Tr*Cel7A on filter paper substrate was processive with an average weighted velocity (time basis) of 0.25 nm s^−1^±0.16 (s.d.) obtained from a total of *N*=180 traces from 64 separate enzymes at 21 °C. The velocity distribution ranges from slow to fast runs spanning 0.1 nm s^−1^ to 0.8 nm s^−1^ (Fig. 2a). Most traces were 30–60 nm in length with a few as long as 150 nm. A smaller study was also carried out on *Cladophora*-derived native cellulose I (*N*=68 from 17 enzymes). In this study, an average weighted velocity, based on trace length (time basis) of 0.25 nm s^−1^±0.35 (s.d.) was observed with velocities 0.05 up to 2.5 nm s^−1^ (Fig. 2b). Motility trajectories on both substrates were generally straight and some contained pauses. Traces terminated by reaching the end of our 10 min measurement window, exiting our detector zone, stalls, or by unbinding. A closer look at the motility on filter paper revealed steps with a fundamental size of 1.3 nm. Step and dwell distributions were extracted through a step-finding algorithm described in the Methods. Gaussian fits captured the overall step size distribution when multiples of 1 × and 2 × the fundamental step size were included for both positive and negative steps (Fig. 3a). Positive and negative fractions represented 68% and 32% of the population, respectively. Distributions of dwell times between steps averaged 1.6 s±0.05 (s.e.m.), yet persistent dwells as long as 10 s were observed. A double exponential fit to the dwell time distribution resulted in time constants of 1.89 s (16%) and 0.52 s (84%; Fig. 3b).

The relationship between step size and dwell time revealed that positive steps were associated with slightly longer dwells than negative steps (Fig. 3c). Particularly straight and fast traces yielded step distributions generally lacking negative steps (Fig. 3d,e).

**Figure 4:**
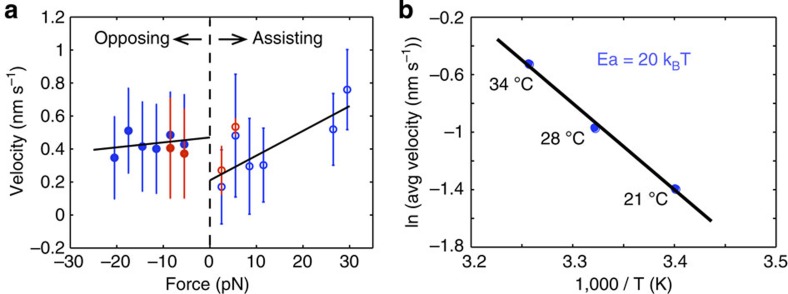
Force and temperature dependence of velocity. (**a**) The force velocity relationship for wt*Tr*Cel7A. Opposing loads (negative) appear to have little effect up to 20 pN while assisting loads (positive) increase velocity. Full data set (blue, *N*=15666 opposing and *N*=5939 assisting) and an axial trace subset (red, *N*=5855 opposing and *N*=208 assisting), in which the force vector is within 18.5° of the cellulose axis. Error bars represent standard deviation (s.d.). (**b**) An Arrhenius fit of wt*Tr*Cel7A motility data from 21 to 34 °C, using average velocity (cellobiose units per s) as a rate, yielding an activation energy of 20 k_B_T (49.8 kJ mol^−1^). The averages are found from *N*=76 (21 °C), *N*=47 (28 °C), *N*=52 (34 °C).

Our measurements included loads generally assisting and opposing motility. Opposing loads (negative axial force) do not appear to significantly affect velocity for loads up to 20 pN, suggesting a stall force greater than 20 pN. The velocity is calculated as the slope of a time trace over a 5-s window and is calculated at each point along a trace. Although a handful of traces appeared to have a stall profile, a formal stall force was not determined as the opposing force velocity profile remained relatively flat. Assisting loads indicate an apparent increase in translocation velocities, nearly doubling for forces approaching 25 pN (Fig. 4a). Note that lines included in Fig. 4a simply highlight the general trend of the force velocity data and not necessarily the precise shape of the relationship. The velocity increase is due to a 0.2-s decrease in dwell time, where apparent long dwells are reduced (Supplementary Fig. 1a). A moderate increase in larger steps is also observed, although it should be noted that the fundamental step size as well as the percent of negative steps remains unchanged. Visualization of these results can be found in Supplementary Fig. 1.

### Motility characterization of isolated CD

To separate the function and behaviour of the CD and CBM from the behaviour of the full enzyme, we expressed isolated CD as well as isolated CBM with a partial linker. In the case of isolated CD, a biotin tag on the N-terminal served as a handle for tethering the domain to the bead in a similar assay geometry to the full enzyme (Fig. 1b), whereas an N-terminal His-tag was used in the case of isolated CBM (Fig. 1c).

Strikingly, motility measurements with isolated CD revealed almost identical stepping and processive motion as the full enzyme (Fig. 3a,e). Slightly shorter dwell times (1.2 s) are most notable when associated with negative steps (Fig. 3b,c). In addition, a double exponential fit of the dwell time distribution shows a shift to a higher preference for the faster step (1/*k*_1_=0.21 s (94%) and 1/*k*_2_=1.25 s (6%)) for isolated CD. This suggests the CD is fully and independently capable of translocation mimicking the full enzyme.

### Activity measurements of wt*Tr*Cel7a and isolated CD

Although SM behaviour is unchanged, it is noted that bulk activity assays were challenging and required implementation of multiple approaches. These assays, specifically mass spectrometry analysis of cellobiose product and fluorescence decoration of fibres, indicate an apparent reduction of activity in CD that is 50 times lower than wt*Tr*Cel7A. The standard Filter Paper Assay was also used to measure activity but the detection limit was insufficient to measure the activity of isolated CD. All three activity assays are highlighted and the data provided in Supplementary Note 1 and Supplementary Figs 2–4, respectively.

Heterologous expression of CD in *E. coli*, which can produce misfolded or inactive proteins^18^, and the lack of pyroglutamic acid^19^ at the N-terminus of the CD, because of the biotin carboxyl carrier protein (BCCP) tag, may contribute to this lack of apparent activity. However, mass spectrometry results show the expression of active enzyme (CD) in *E. coli* and fluorescent binding studies suggest that the observed decrease in apparent activity for CD is almost exclusively due to a lack of binding facilitated through the CBM. Note that we detect no difference in the motility traces so any effects from a lack of pyroglutamic acid are likely to occur upstream during motility commitment. Rather, we believe the large differences observed in activity here are due to the lack of CBM, which increases the local concentration of CD near the substrate and the motor's commitment to degradation. CBM function is rescued in our CD experiments when the bead is actively brought near the surface to bind. It is conceivable that the lack of the CBM could allow for large differences in apparent activity at the bulk scale, whereas processive motility remains unchanged as observed at the SM level. This type of discrepancy is seen in other motor systems such as ClpXP, in which a fourfold difference in commitment to degradation is observed when comparing bulk solution to SM results^20^. In addition, any concern regarding the analysis of inactive motor is eliminated in SM experiments where only motility from active motors is recorded. Our observations are supported by Igarashi^6^ and Nidetzky^21^ who report similar activities of absorbed *Tr*Cel7A and *Tr*Cel7A_core_ (CD) while also noting, in the case of Igarashi *et al*, that at least a 10 × higher concentration of CD is needed to observe binding.

### CBM studies

Loaded SM measurements on tethers formed directly through isolated CBM domain binding revealed no motility and an average bond lifetime of 1.7 s±0.27 (s.e.m.), which is consistent with the non-productive off rate reported by Shibafuji^13^. This average represents lifetimes at a range of forces, with an average (and median) force of 7.1 (and 5.2) pN±0.95 (s.e.m.). Solution-based binding through Biacore on substrate and motors identically prepared to those used here reveals an off rate of 0.32 s^−1^, corresponding to a bond lifetime of 3.1 s, slightly longer than the SM dwell time. A CBM activity assay (detailed in the Methods section and Supplementary Note 1) reveals that a significant fraction of our expressed CBM actively bind cellulose.

### Temperature dependence of wt*Tr*Cel7a

Although the described studies were carried out at 21 °C, *Tr*Cel7A performance is optimized near 50 °C (ref. 22). A temperature of 50 °C was not used because of instrument limitations, however, we did track wt*Tr*Cel7A at temperatures from 20 to 34 °C. Measurements at elevated temperatures can reveal activation barriers to mechanical cycle^23^,^24^. As expected, the velocity of wt*Tr*Cel7A increases with an elevating temperature (0.25, 0.4 and 0.6 nm s^−1^ at 21, 28 and 34 °C, respectively; Fig. 4b). Step sizes remain constant while dwell times are reduced (1.6, 1.4 and 1.2 s, respectively). Here we used an assay with the motor bound directly to the bead (no DNA tether) because of increased noise at elevated temperatures. To ensure consistency between this assay and the DNA tethered assay at 21 °C, the temperature-dependent results were compared with a smaller study incorporating the DNA tether, in which the temperature was only elevated to 28 °C. The observed rates are consistent within error.

## Discussion

This optical tweezers-based motility assay permits direct monitoring of *Tr*Cel7A motion revealing steps, dwell times and energetic barriers associated with cellulose degradation. The 1.3-nm fundamental step size identified here suggests the motility cycle is tied to processing of a single cellobiose unit (1 nm). Although we believe a single step underlies the dominant preferred cycle, *Tr*Cel7A is also able to step in multiples of this unit, move backwards and pause. Backstepping and pausing, common in other motors^25^,^26^,^27^,^28^,^29^, may allow *Tr*Cel7A to negotiate roadblocks, including those created by its own CBM and LDs, and re-set motility.

Our study reveals processivity of isolated *Tr*Cel7A motors is at least 50 steps (50 nm). Here we conservatively analysed our traces, only considering segments with no obvious interruption in motility. Our processivity results, in which *Tr*Cel7A stays bound for 1.5 min or longer, are consistent with ‘productive binding' dwell times reported earlier^14^,^15^. Given the relative insensitivity of wt*Tr*Cel7A to load, these results suggest the CD domain is sufficient for maintaining a tight grip during active motility.

Our temperature study yields an activation barrier of 20 k_B_T (49.8 kJ mol^−1^) to stepping. This assumes a velocity in which one catalytic event is equal to one cellobiose unit (∼1 nm step). Thus, the velocity is treated as a rate without a unit length correction (Fig. 4b). This result is similar to the rate-limiting barrier of 15.5 kcal mol^−1^ (∼60 kJ mol^−1^) found by Knott and co-workers in their 2014 computational study^30^. From a thermodynamic analysis of the cellulose degradation process, a mere quarter of this activation energy is available from the glycosidic bond, 5 k_B_T or 12.5 kJ mol^−1^ (refs 6, 31). Crystalline cellulose contains hydrogen bonds within and between adjacent polymer strands, which may provide more than half this barrier, 13.86 k_B_T or (4 bonds*3.38 k_B_T or 34.3 kJ mol^−1^). Combining the hydrogen bonds with the glycosidic bond energy represents 94% of the barrier. An additional 2.32 k_B_T (5.73 kJ mol^−1^) is available from solvation of cellobiose. From a work perspective, we can conservatively estimate that *Tr*Cel7A can step 1 nm against a 20-pN load, which is 20 pN*nm or ∼5 k_b_T, equal to virtually all of the energy available from the glycosidic bond. Both the activation barrier result and measured work suggest *Tr*Cel7A uses energy in addition to the glycosidic bond such as contributions from cellulose decrystallization, hydrogen bonding and cellobiose solvation. In this analysis, we consider the energies available in the context of the initial and final state of each cycle of processive steps such that the order of energy usage from these potential sources is not determined or dictated. In addition, the flat profile of the force-velocity curve for opposing load suggests that the rate-limiting steps in a cycle are non-mechanical (biochemical)^32^,^33^.

Comparing wt*Tr*Cel7A, isolated CD and CBM constructs parses the role of each of the various sub-domains. The CD fully captures step size and dwell time distributions of wt*Tr*Cel7A motility, establishing that the motility cycle mechanism is wholly contained within the CD unit. This indicates that the CD is independently capable of the translocation exhibited by the full wt*Tr*Cel7A enzyme. However, the shorter dwell times of the isolated CD construct suggest that the presence of the CBM and LDs may slightly impede motility. At the same time, CBM bond lifetimes (1.7 s), while consistent with wt*Tr*Cel7A dwell times (1.6 s), are sufficiently weak in comparison to the performance of the wt*Tr*Cel7A motor. This indicates that the CBM is likely designed to avoid impeding motility while also increasing substrate adsorption. CBM bond lifetimes are similar to those found in solution through Biacore (3.1 s) and consistent with reported non-productive binding times (1.16 s)^13^.

Given the relative processivity of the CD it is curious why *Tr*Cel7A maintains its ‘ball and chain' partner. The presence of the CBM suggests binding enhancement is needed for initial commitment to motility, a rate-limiting step, where a free cellulose end is found and threaded into the CD. Actively placing a CD functionalized bead atop a cellulose strand, artificially associating the two as in our tweezers-based assay, was sufficient to initiate motility. Strong initial binding to cellulose by the CD itself might aide in initiating motility, but interfere with productive stepping. Sequestering these tasks across separate domains, through a flexible linker, with similar stepping and off rate times achieves both goals while minimizing physical interference between conflicting desires to bind, negotiate roadblocks and move. The cellulose/cellulase system in many ways parallels other degradation machinery and strategies found in systems such as in protein degradation by ClpXP^20^,^32^ and collagen by collagenases^34^, both of which play significant roles in balance and regulation of natural systems.

Leveraging this and other SM assays with functional mutation of motor domains holds much promise in revealing cellulose degradation mechanisms. In contrast to this SM assay, Igarashi measured higher velocities using highly crystalline algae-derived cellulose under conditions of higher motor concentration (30-fold greater)^7^ supporting previous studies that suggest subtle variations in substrate and motor–motor interactions may significantly enhance motility^12^,^35^,^36^,^37^. Despite high spatial resolution of the atomic force microscopy, individual steps were not observed. The observed velocity increase with assisting loads seen here suggests multiple motor interactions, as seen in by Igarashi *et al*^7^, may substantially enhance degradation rates. Both Igarashi's and the present study report a spread of velocities while some individual traces exhibit relatively straight trajectories. The spread in velocities can be indicators of heterogeneity in types of motility, stochastics of individual motors and substrate inhomogeneity while the level of uniformity within individual traces can occur from a cycle containing multiple steps of similar rates in individual motor or from multiple motors working in concert. Future work focused on cellulose degradation systems with multiple enzymatic components will investigate various processing strategies of cellulose substrates and synergistic interactions between cellulase cocktail component enzymes. Ultimately, processes that integrate biological strategies with industrial processing of cellulose as an energy source have much promise.

## Methods

### Materials

Sulfo-SMCC crosslinker (Thermo Scientific—22622), 1,010 bp DNA functionalized with appropriate end groups (primers specified in the following methods), 1.25 μm streptavidin polystyrene beads (Spherotech—SVP-10-5), 0.75 μm polystyrene beads (Spherotech—PP-08-10), 1.0 μm carboxylated polystyrene beads (Polysciences—08266), biotinylated BSA (5 mg ml^−1^, received as a gift from Ted Feldman at Harvard University), BSA (Sigma—A3059), 50 mM acetate buffer (pH 4.9), PBS (1 × , pH 7.4), Whatman Grade 1 Filter Paper (Sigma—WHA1001110), *Trichoderma reesei* cellulase mixture (Sigma—C2730) and deionized (DI) water were used.

### Enzyme purification (wtTrCel7A)

wt*Tr*Cel7A was purified from a *Trichoderma reesei* cellulase mixture (Sigma C2730) using step-elution ion chromatography^17^. First, 450 μl cellulase mixture was buffer exchanged to 10 mM TEA-HCl (pH 7.6) using 6 × Bio-Rad P30 chromatography columns according to the columns' protocol. Purification was then carried out using a Q-Trap ion chromatorgraphy column (GE Healthcare – 17-1153-01). The column was first washed with 5 column volumes (5 ml) of 20 mM TEA-HCl (pH 7.0) at a rate of ∼1 ml min^−1^. Note that flow through the column is promoted through a pressure differential created by applying a vacuum to the collection container. The column was then washed with 5 column volumes (5 ml) of 0.1 M NaCl in 20 mM TEA-HCl (pH 7.0) and again washed with 5 ml of 20 mM TEA-HCl (pH 7.0). Three hundred microlitres of the cellulase sample is then loaded into the top of the column. The column was washed with 4 ml of 20 mM TEA-HCl (pH 7.0). The other enzymes in the sample were then eluted by applying 2 ml 0.1 M NaCl in 20 mM TEA-HCl (low salt solution) to the column. Finally, the desired *Tr*Cel7a was eluted by applying 1.6 ml of 0.33 M NaCl in 20 mM TEA-HCl, pH 7.0 (high salt solution) to the column. Purity was confirmed using SDS–polyacrylamide gel electrophoresis (Supplementary Fig. 6), where *Tr*Cel7A is ∼64–66 kDa and accounts for 55–60% of the cellulase mixture^10^,^17^. Purified samples were buffer exchanged to acetate buffer with 10% glycerol, diluted to 0.4 mg ml^−1^ aliquots and stored at −80 °C for future use.

### Enzyme expression and purification (isolated CD and CBM)

CBM and the CD were both expressed in GC5 competent *E. coli*. The CBM includes 9 aa of the LD with a His tag at the N terminus (His_6_-TTTGSSPGP-TQSHYGQCGGIGYSGPTVCASGTTCQVLNPYYSQC). The CD has a biotin tag, via a His_6_ tag and BCCP link, at the 11th amino acid (His) on the N terminus. No LD is present in the CD construct.

### Expression plasmids

DNA fragments encoding the LD sequence with the CBM region and the CD region were amplified by PCR using primers containing appropriate restriction sites for fusion and insertion into the pUC 57 plasmid to create entry clones of the constructs. The primers used are as follows: for the CBM, forward primer: 5′-CCCGACCCAGAGCCATTAT-3′ and reverse primer: 5′-CACTGGCTATTAATACGGGTTCAG-3′, and for the CD, forward primer: 5′-CGATACCCCTGTGCATTGTGG-3′ and reverse primer: 5′-AGTTCGCATCAATCACCACG-3′. The PCR conditions were 30 cycles of denaturation at 94 °C for 1 min, annealing at 50 °C for 2 min and extension at 72 °C for 30 s with the final extension step run for 5 min. The sequence encoding the CBM with linker was restricted with *Nde*I and *Xho*I (Promega) and sub-cloned between the restriction sites of pET28b expression vector, enabling fusion of the polypeptide His_6_ tag. In the case of the CD, the entire fragment encoding the CD of *Tr*Cel7A with a BCCP subunit was subcloned into *Nde*I and *Xho*I sites of the pET28b expression vector with fusion of the polypeptide His_6_ tag. The recombinant plasmids were then transformed in GC5 (Sigma-Aldrich) competent *E.coli* cells. Kanamycin-resistant colonies were isolated and their nucleotide sequence verified by DNA sequencing (First base).

### Enzyme production and purification

Overnight pre-cultures of the transformants (2.5 ml) were inoculated into 100 ml of lysogeny broth (LB) medium containing 50 μg ml^−1^ of kanamycin and shaken at 37 °C and 120 r.p.m. Protein expression was induced by adding 1 mM of isopropyl-β-D-thio-galactopyranoside, when the OD_600_ reached 0.4–1.0. After induction, the transformants were grown under the same conditions for 6 h (CBM and CD) to produce proteins. After cultivation, cells were collected by centrifugation at 5,000*g* for 10 min and gently disrupted by the use of B-PER bacterial extraction reagent (Thermoscientifc) according to the manufacturer's instruction. Two volumes of 100 mM sodium acetate buffer (pH 4.0) was added to remove the *E.coli*-derived proteins. After 1 day, the cell suspension was centrifuged at 18,000*g* for 20 min. The resulting soluble extract was purified with HisTrap FF Ni^2+^-NTA affinity column (GE Healthcare). The supernatant containing the purified enzyme was dialysed by ultrafiltration (PBGC membrane, Millipore) with 10 mM acetate buffer to pH values allowing for protein stability. The purified enzymes were frozen and stored at −20 °C. Purity of the proteins was confirmed using SDS–polyacrylamide gel electrophoresis (Supplementary Figs 7 and 8). Protein concentrations were determined using Nanodrop 1000 (Thermoscientific).

### Bead functionalization

All proteins were tethered to polystyrene beads with a 1,010-bp DNA linker with the appropriate functionalizations. The 1,010-bp DNA linkers were created using PCR and the M13mp18 plasmid template. All primers were ordered from Integrated DNA Technologies (IDT). In the case of wt*Tr*Cel7A, one 5′ biotinylated primer (forward, 5′-biotin-TATTGCGTTTCCTCGGTTTC-3′) and one 5′ thiol-functionalized primer (reverse, 5′-thiol-TTGAAATACCGACCGTGTGA-3′) were used with the M13mp18 template. One 5′ amine primer (reverse, 5′-amine-TTGAAATACCGACCGTGTGA-3′) and one 5′ digoxygenin-functionalized primer (forward, 5′-Dig-TATTGCGTTTCCTCGGTTTC-3′) were used for isolated CD and isolated CBM. After PCR, the amine-functionalized end of the tethers for isolated CD were crosslinked to ½ anti-biotin antibody (described later) while tethers for isolated CBM were crosslinked to anti-His antibody (Genscript—A000186-100), using EDC chemistry. Similar DNA linkers have been used in the lab previously^32^,^38^.

In the case of wt*Tr*Cel7A, enzyme was tethered to 1.25 μm streptavidin polystyrene beads (Spherotech—SVP-10-5) in three steps to achieve approximately one enzyme per bead. First, a wt*Tr*Cel7A sample was reacted with sulfosuccinimidyl 4-(N-maleimidomethyl)cyclohexane-1-carboxylate (sulfo-SMCC) crosslinker in PBS on a rotator at room temperature for 30 min. The reaction targets the approximately eight surface lysines found on the CD. Following incubation, excess crosslinker was removed using a Biorad P30 chromatography column. The resulting proteins were then bound to the thiol-functionalized end of a 1,010-bp DNA linker via the sulfo-SMCC crosslinker by incubating the components at room temperature in PBS (1 × , pH 7.4) for 30 min, on a rotator. Finally, functionalized beads were created by mixing biotinylated linker-bound enzyme and biotinylated BSA (1:10 ratio), with a final enzyme concentration of ∼5 μg ml^−1^ (∼78 nM TrCel7A), and 1.25 μm streptavidin polystyene beads in acetate buffer. The bead solution is incubated on a rotator at 4 °C for 45 min. After incubation, beads were washed by centrifuging for 2.5 min at 9,400*g*, aspirating, and re-suspending the pellet in 50 mM acetate buffer (pH 4.9) to remove unreacted biotinylated BSA and enzyme.

CD functionalized beads were created by tethering CD to 1.0 μm anti-Dig-coated polystyrene beads in one step. The protein, expressed with a BCCP biotin tag is mixed with picomolar concentrations of 1,010 bp DNA tethers (functionalized with Dig at one end and ½ anti-biotin at the other), anti-Dig beads and BSA (0.125 mg ml^−1^ final concentration) in PBS. After incubation on a rotator at 4 °C for 45 min, the beads are washed and the buffer replaced with 50 mM acetate buffer (pH 4.9) three times by centrifuging the sample for 4 min at 8,000*g* in order to remove excess and unreacted CD and DNA. The ½ anti-biotin used to functionalized the DNA tether is prepared as described below.

CBM-functionalized beads were created by tethering CBM to 1.0 μm anti-Dig-coated polystyrene beads in one step. The protein, expressed with a His_6_ tag, is mixed with picomolar concentrations of DNA tethers (functionalized with Dig at one end and anti-His at the other), anti-Dig beads and BSA (0.125 mg ml^−1^ final concentration) in PBS. After incubation on a rotator at 4 °C for 45 min, the beads are washed and the buffer replaced with 50 mM acetate buffer (pH 4.9) three times by centrifuging the sample for 4 min at 8,000*g* in order to removed excess and unreacted CBM and DNA.

### Partial reduction of anti-biotin antibody

Anti-biotin antibody is obtained from Sigma-Aldrich (B3640) and dissolved into reaction buffer (20 mM sodium phosphate, 0.15 M NaCl, 5 mM EDTA at pH 7.4) to a concentration of 10 mg ml^−1^. A 19 mg ml^−1^ solution of 2-MEA in reaction buffer is also prepared. Two microlitres of 2-MEA solution is then mixed with 10 μl anti-biotin antibody solution and incubated for 90 min at 37 °C. Reduced antibody is then purified using a Bio-Rad P30 chromatography column. The methods described here are the same as those used by Das *et al*.^38^ The resulting ½ anti-biotin antibody is linked to the amine functionalized end of DNA tethers using Sulfo-SMCC crosslinker.

### Filter paper preparation

The substrate used in these studies, except when specifically specified as *Cladophora*, is Whatman Grade 1 Filter Paper (Sigma—WHA1001110) and contains 99% cellulose. To generate the desired fibres, small 1 mm^2^ pieces were cut from a single filter paper sheet. Approximately 20 mg of filter paper was wetted with a few drops of DI water in a tissue homogenizer and homogenized for 5 min. The substrate solution was then diluted to a mixture of 2 mg substrate per ml with 50 mM acetate buffer (pH 5.0). The substrate was then subjected to mechanical processing through repeated sonication, vortexing and shearing/mixing by passing the solution through a 16-gauge syringe. The substrate was stored at 4 °C until use.

### Cladophora cellulose preparation and characterization

Highly crystalline, triclinic cellulose Iα from *Cladophora* sp. (*Cladophora glomerata*) filamentous green algae was harvested from fresh water Lake Mendota (Madison, WI, USA) and adjoining water-bodies connected by the Yahara river^39^,^40^. Recovered algae was washed extensively with deionized water to remove contaminants before long-term storage at −20 **°**C. The cleaned algae fibres were bleached for 30 min at room temperature by immersing 5–15 g of algae (on dry weight basis) in 100 ml of 10% acetic acid solution containing 20 g sodium chlorite. The solution was diluted to 1 l with deionized water, incubated at 60 **°**C in a hot water bath for 3 h and subsequently washed to neutral pH with deionized water. The fibres were then incubated twice overnight in a 1 l solution of 4% sodium hydroxide at 60 **°**C to facilitate removal of non-cellulosic polysaccharides. The recovered samples were then washed to neutral pH and incubated with 250 ml of boiling solution of 5% hydrochloric acid for 15 min and left incubated in the same solution overnight at room temperature to facilitate hydrolysis and removal of amorphous cellulose. The samples were washed extensively with deionized water till neutral pH the next day to obtain highly crystalline cellulose fibrils suspension slurry. The cellulose slurry was either stored at 4 **°**C in the presence of sodium azide or lyophilized for long-term storage and further characterization by X-ray diffraction, Raman spectroscopy and carbohydrate composition analysis. The algal cellulose extraction conditions were varied (Supplementary Table 1) to maximize total crystalline cellulose content (Supplementary Fig. 9a) as estimated using standard carbohydrate detection and quantification methods^41^. In addition, non-cellulosic polysaccharide contamination was verified to be minimal for all samples (Supplementary Fig. 9b) with crystalline cellulose content for samples used in this study to be 96.2±5.7% (dry weight basis).

Pelletized lyophilized cellulose samples (100–150 mg; dry weight per pellet) were analysed on a Bruker MultiRam spectrometer (Bruker Instruments Inc.) at the USDA-FPL. Data were collected using a 600-mW Nd-YAG laser with 512 scans per sample. Bruker's OPUS software was used to determine peak positions and analyse Raman spectral data. Data were normalized at 1,096 cm^−1^ wavenumber shift to facilitate comparison between different samples^42^. Raman spectroscopy analysis confirmed the algal cellulose allomorph type to be predominantly Iα in content as indicated by the OH stretching vibrations resultant shoulder around wavenumber 3,240 cm^−1^, which is absent in Iβ (refs 37, 43, 44). Other characteristic Raman spectral features for algal cellulose Iα compared with plant-derived cellulose Iβ control (Avicel) are also shown in Supplementary Fig. 10. X-ray diffraction analysis (Supplementary Fig. 11) was also carried out on dried algal cellulose Iα fibres to determine total cellulose crystallinity of >92% based on the Segal method^37^. X-ray analysis confirmed the absence of any regenerated cellulose II allomorph formed during the algal cellulose isolation process.

### Slide preparation

Slides were prepared by first creating an ∼15 μl flow cell using double-sided sticky tape. The flow cell is then loaded through capillary action with diluted cellulose slurry. In all cases the substrate is nonspecifically immobilized on the coverslip surface by allowing the loaded chamber to evaporate until dry in an oven at 85–90 °C (∼1 h). Dried slides were then allowed to cool to room temperature and incubated with a BSA blocking solution before loading the functionalized beads. In the case of wt*Tr*Cel7A and isolated CD experiments, 0.75 μm non-functionalized beads were allowed to nonspecifically adhere to the coverslip surface in an incubation step before BSA blocking. These beads served as fiducial markers allowing for instrumental drift tracking during data acquisition.

### SM data collection

Enzyme-functionalized beads were trapped with a 1,064-nm laser and placed alongside a surface-bound stationary fiber. After position calibration and trap stiffness measurements, the bead was held above the fibre to facilitate binding between the protein and the substrate. Upon binding, the bead was centred by an automated two axis piezo stage centring routine and loaded with up to 25 pN of force through piezo stage offset motions. Bead position was recorded at 3 kHz to track motility records as long as 10 min. To account for instrumental drift during the measurements, the smaller non-functionalized fiducial beads were tracked simultaneous with motility records using an Andor Ixon camera to acquire synchronized images at ∼4 Hz. In the collection of CBM bond lifetime data, instrumental drift was not tracked because of the shorter acquisition times and the nature of the data being collected. Thus, fiducial beads were left out of the assay.

Experiments were carried out at 21 °C except for temperature-dependent studies. For experiments at elevated temperatures (28 and 34 °C), the temperature-controlled box surrounding the microscope was equilibrated to the set temperature before data acquisition. Temperature-dependent data sets include DNA tethered wt*Tr*Cel7A (28 °C) as well as enzyme bound directly to the bead using biotinylated succinimidyl ester (21, 28 and 34 °C).

### Data analysis

Given the low velocity of the *Tr*Cel7A motor, and long experiment acquisition time (∼10 min), we developed a method to appropriately handle sample drift. Our strategy included tracking a stationary fiducial bead so that its position could be removed from the baseline of the motor-bead position traces to yield the true enzyme trajectory. To do this, we employed a cross correlation video tracking algorithm and time-synchronized our video with motor-bead tracking by starting both acquisitions simultaneously. Drift data were then smoothed and subtracted from the motor-bead tracking data.

Data were collected at a 3-kHz sampling frequency in all experiments and decimated to 20 Hz during further analysis. Nanometre position and piconewton force values were found using calibration data and trap stiffness. Custom MATLAB scripts were used to calculate bond lifetimes, velocities and local force-velocity information. The step finding script was modified from a previous version within the lab and based on a sliding Student's t-test that detects the edges of each step to prescribe a dwell and allows for varied step sizes^45^. A dwell was defined as a period of constant position between bursts of motion (steps). Specifically, an enzyme is said to be in a dwell if the change in the moving average of position is less than 0.9 nm (the minimum step-size threshold). This threshold was chosen as a result of step finding optimization and the 1-nm step resolution controls. It was determined that the first bin of the dwell distributions is an artifact of the analysis and is not representative of actual behaviour at those times. Thus, dwell distribution fits ignore the first bin. It is also noted that step analysis for each trace was visually inspected for accuracy. The same analysis conditions were used for all traces in a data set. As such, step finding was not optimal for all traces, for example, a trace with larger or smaller noise than average, or those traces with very low velocity. In these cases, step analysis was not always sufficiently accurate. If more than one step was obviously missed or if noise was too large, resulting in an apparent large number of steps, the traces were not used for step and dwell analysis.

Force changes during the motility trace as the bead is pulled within the fixed trap. The force was evaluated along a trace using bead position relative to the trap centre over 5 s windows. The associated average velocity over the same time period was then evaluated using a linear fit of the drift-adjusted time trace.

### Activity: mass spectrometry

To investigate enzymatic activity, mass spectrometry was used to analyse the cellobiose product (molecular weight=342.3) of both wtTrCel7A and isolated CD samples (1 mg ml^−1^ enzyme) that were incubated with filter paper for 68 h at room temperature. In preparation for mass spectrometry, product from isolated CD, only filter paper-negative control (no enzyme), and wtTrCel7A were diluted 0.6:10, 1.2:21.2 and 1:300, respectively, into 95.5% acetonitrile (CH_3_CN) in water. Standards were prepared by creating a 100 μM solution of cellobiose (Sigma) in Millipore water and diluting to appropriate concentrations using 95.5% acetonitrile (CH_3_CN) in water.

Samples were analysed using LC-MS/MS at the Vanderbilt Mass Spectrometry Core Laboratory on a Water Acquity UPLC system (Waters), made up of a binary solvent manager, refrigerated sample manager and a heated column manager. Tandem mass spectrometric detection was performed using a TSQ Quantum triple quadrupole mass spectrometer (Thermo Scientific) equipped with an Ion Max API source, and ESI probe, and a 50-μm ID stainless steel capillary. Experiments were performed by Assistant Director, Dr Wade Calcutt, using his procedure below.

A Sequant ZIC-cHILIC PEEK analytical column (2.1 × 150 mm^2^, 3.0 μm particle size, Merck) was used for all chromatographic separations. The mobile phases consisted of 0.2% AcOH and 5 mM NH_4_OAc in (A) H_2_O/CH_3_CN (9:1) and in (B) CH_3_CN/MeOH/H_2_O (90:5:5). A flow rate of 300 μl min^−1^ for 15 min was used with sample volumes of 10 μl. Mass spectrometry analyses were in positive ion mode with a scan time of 100 ms and scan width of 0.5 Da. The calibration curve was created using the LC-MS/MS peak area against analyte concentration for cellobiose standards from 1 to 150 μM.

### Activity: fluorescence decoration of fibres

Binding activity was tested by observing specific decoration of both wt*Tr*Cel7A and CD to immobilized filter paper substrate. Both wt*Tr*Cel7A and isolated CD were labelled with TAMRA(5)-succinimidyl ester (se) fluorophores overnight in PBS by reacting the succinimidyl ester end of the fluorophore with surface lysines on the CD. The buffer was then exchanged to 50 mM acetate buffer (pH 4.9) and excess fluorophore removed using three P-30 column washings (Bio-Rad) in series. The concentration of each solution was found using a NanoDrop and samples diluted to known molarities. Slides with immobilized cellulose (filter paper), prepared as described in the Methods section of the manuscript, were loaded with labelled enzyme of various concentrations.

### Activity: filter paper assay

In these studies, an enzymatic activity assay using our standard filter paper substrate was conducted where reducing sugars are reacted with dinitrosalicylic acid to form a spectroscopically detectable product. This assay was scaled down to a 7.5 μl reaction volume from the original 1987 procedure^46^ and a small volume 96 well plate procedure (as described in the review by Dashthan *et al*.^47^). To do this, 7.5 μl of enzyme at 1 mg ml^−1^ (or cellobiose standard at appropriate concentration) was added to a microcentrifuge tube containing 1/16 of a filter paper disk created using a 3-ring hole punch. The sample was allowed to incubate at room temperature for 50 h. At the end of the incubation time, 15 μl of dinitrosalicylic acid solution was added to the sample and incubated for 25 min at ∼100 °C to generate the colour change associated with its reaction with reducing sugars (cellobiose). After cooling to room temperature, the reducing sugar concentration of each sample is measured by finding the absorbance at 540 nm, using the NanoDrop (Supplementary Fig. 4).

### Activity: CBM

During purification, CBM was purified using a His-tag column. Affinity estimates from wash and resuspension stages of a cellulose-based purification procedure was also used to test the strong binding activity of the CBM, as follows. A custom cellulose column was first created in which a 0.7 ml Eppendorf tube was filled half-way to the 100 μl line with cellulose (cotton linters, Sigma). A 0.634 mg ml^−1^ (106 μM) CBM sample in 50 mM acetate buffer (183 μl total) was added to the tube and rotated at 4 °C overnight. The next morning, to remove excess protein, the sample was centrifuged at room temperature for 15 min at 18,000*g* and the supernatant removed (labelled ‘flow through'). The cellulose was resuspended in 300 μl of 50 mM sodium acetate buffer (pH 4.9) and again centrifuged for 15 min at 18,000*g*. The supernatant was collected and labelled ‘wash'. The wash collects protein exhibiting low binding.

## Additional information

**How to cite this article:** Brady, S. K. *et al*. Cellobiohydrolase 1 from *Trichoderma reesei* degrades cellulose in single cellobiose steps. *Nat. Commun.* 6:10149 doi: 10.1038/ncomms10149 (2015).

## Supplementary Material

Supplementary Supplementary Figures 1-11, Supplementary Table 1, Supplementary Notes 1-2 and Supplementary Reference
