# Supplementary material for: Cellobiohydrolase 1 from Trichoderma reesei degrades cellulose in single cellobiose steps
Source: Nat Commun. 2015 Dec 10;6:10149. doi: 10.1038/ncomms10149 (PMC4682103; doi:10.1038/ncomms10149)
Supplement: Supplementary — Figures 1-11, Supplementary Table 1, Supplementary Notes 1-2 and Supplementary Reference [file ncomms10149-s1.pdf]

SUPPLEMENTARY FIGURES

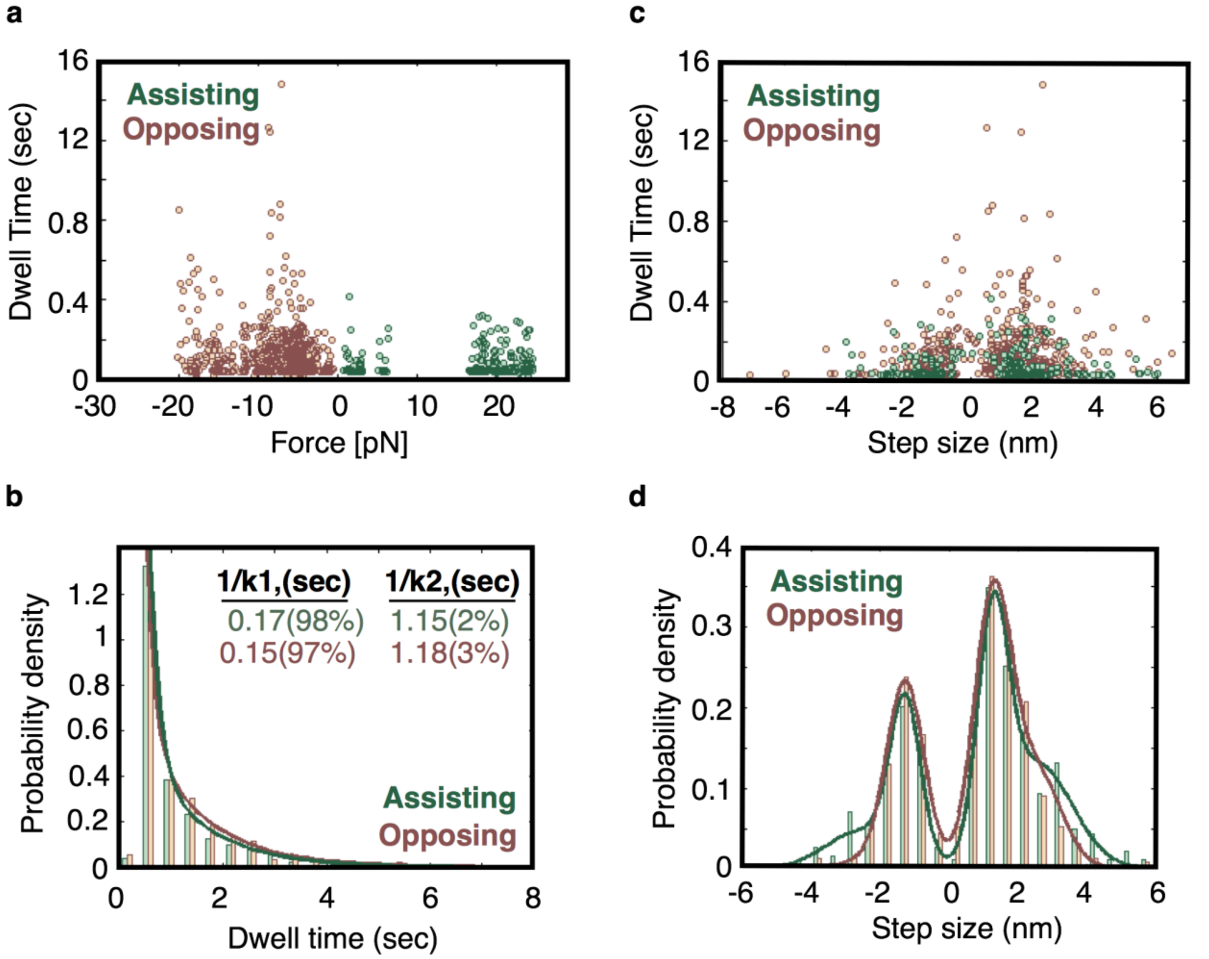

Supplementary Figure 1. Stepping analysis of opposing (brown) and assisting (green) traces of wt*Tr*Cel7A. (a) The relationship between dwell time vs. force for different load types. Note the reduction of long dwells for assisting load motility. **(b)** Dwell distributions fit to a double exponential with the fit values indicated in the figure. **(c)** The relationship between dwell time vs. step size. **(d)** Step size distributions fit to the sum of Gaussian curves based on the fundamental and 2x fundamental forward and rearward steps yield a fundamental step size of 1.3nm.

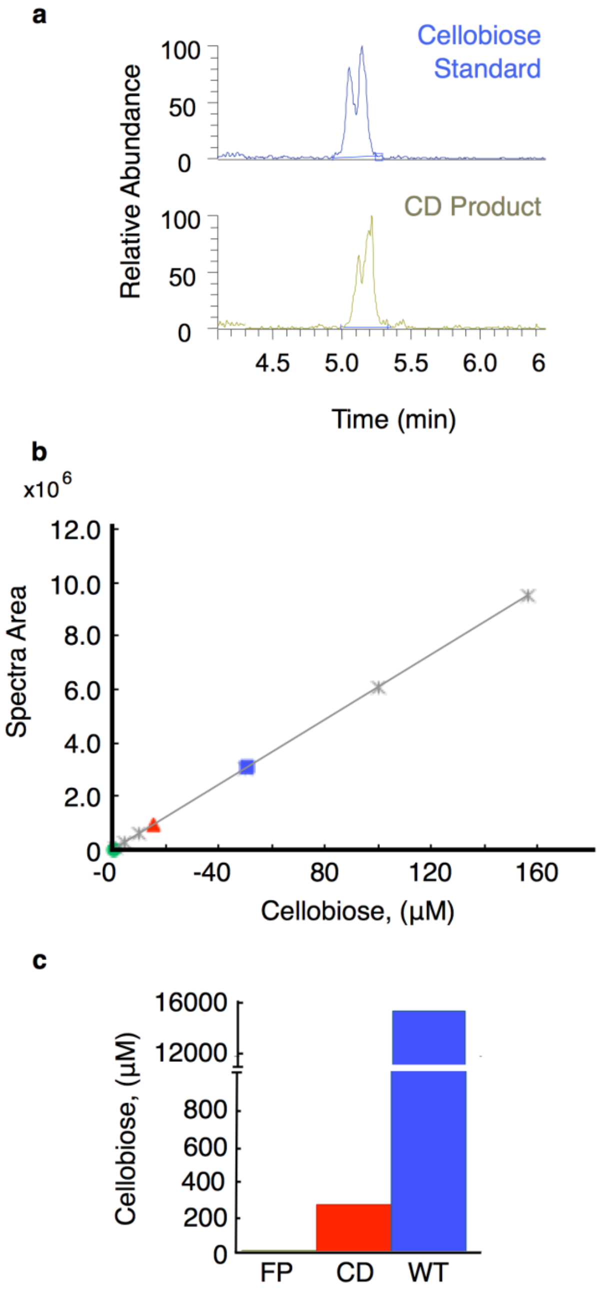

Supplementary Figure 2. Mass Spectrometry results – (a) The spectra show a 1 µM cellobiose standard (Sigma - C7252) peak (top - blue) for comparison to the peak obtained from CD product (bottom - green) demonstrating that enzymatic product is consistent with that of cellobiose. **(b)** Cellobiose calibration curve (grey 'x') and diluted sample product concentration including filter paper control (green circle), CD product (red triangle), and wt*Tr*Cel7A (blue square). **(c)** A comparison of undiluted enzymatic product from the filter paper control (FP), CD, and wt*Tr*Cel7A (WT).

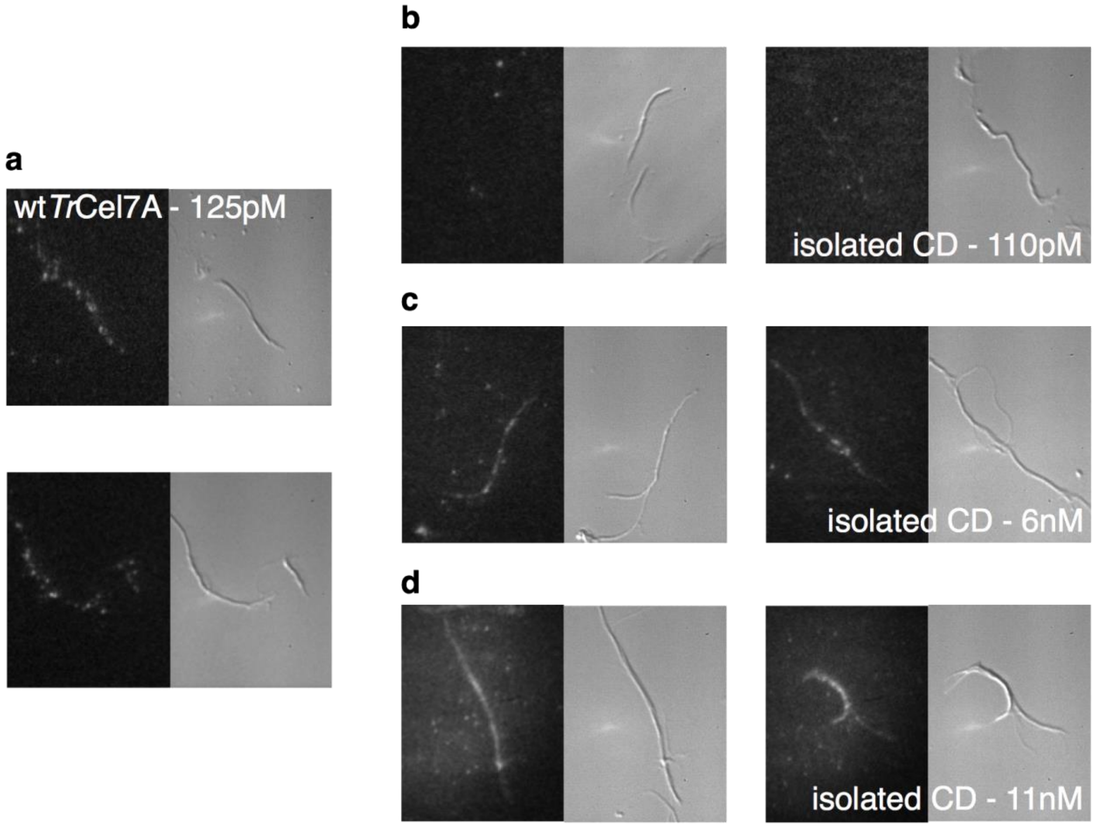

Supplementary Figure 3. Fluorescently labelled protein specifically binds substrate – Fluorescent images and the corresponding bright field (DIC) images show specific binding of TAMRA(5)-se labelled protein to surface bound fibers. The degree of decoration can be compared between two representative pairs of each condition for **(a)** wt*Tr*Cel7A at 125pM, **(b)** CD at 110pM, **(c)** CD at 6nM, and **(d)** CD at 11nm. Similar decoration of 125pM wt*Tr*Cel7A (a) is achieved for 6nM CD (c) which is approximately 50x more concentrated that wt*Tr*Cel7A.

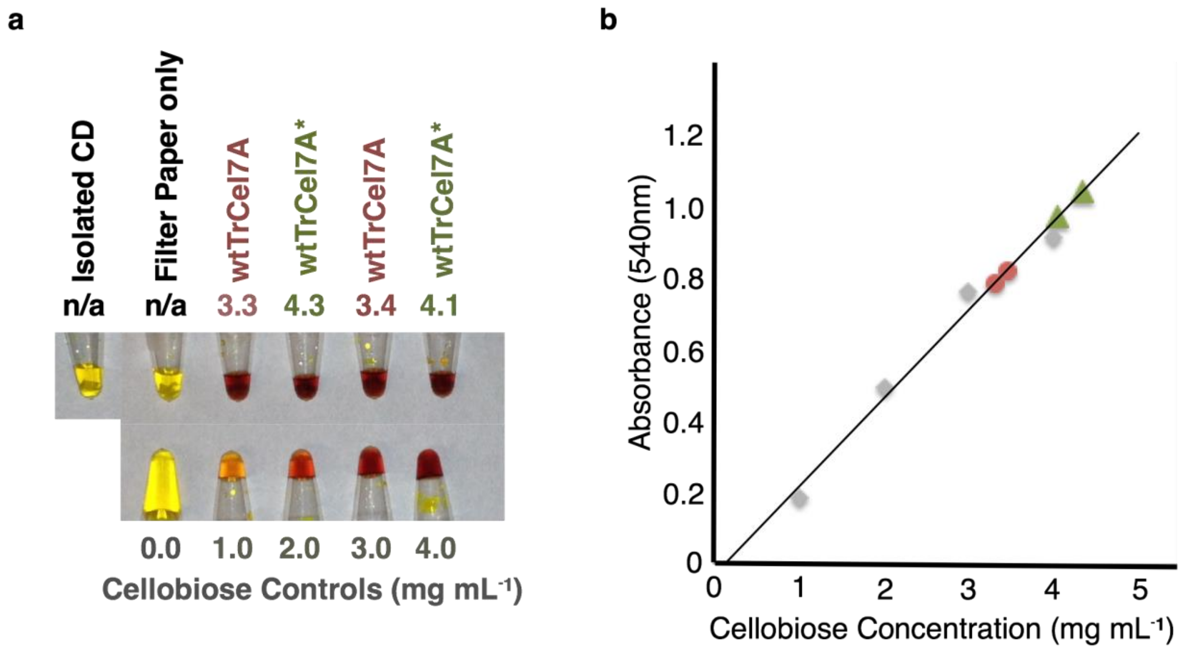

Supplementary Figure 4. Filter paper assay (FPA) – Filter paper assay using DNS to visualize reducing sugar (cellobiose) product generated over 50 hours at room temperature. (A) Visual confirmation of activity by wt*Tr*Cel7a, both with (wt*Tr*Cel7A*) and without (wt*Tr*Cel7A) succinimidyl ester modification and a lack of detectable activity by isolated CD and a filter paper negative control. (B) Analysis of activity measured at 540nm shows high activity of both wt*Tr*Cel7A (red circles) and wt*Tr*Cel7A* (green triangles) with slightly elevated activity for wt*Tr*Cel7A*.

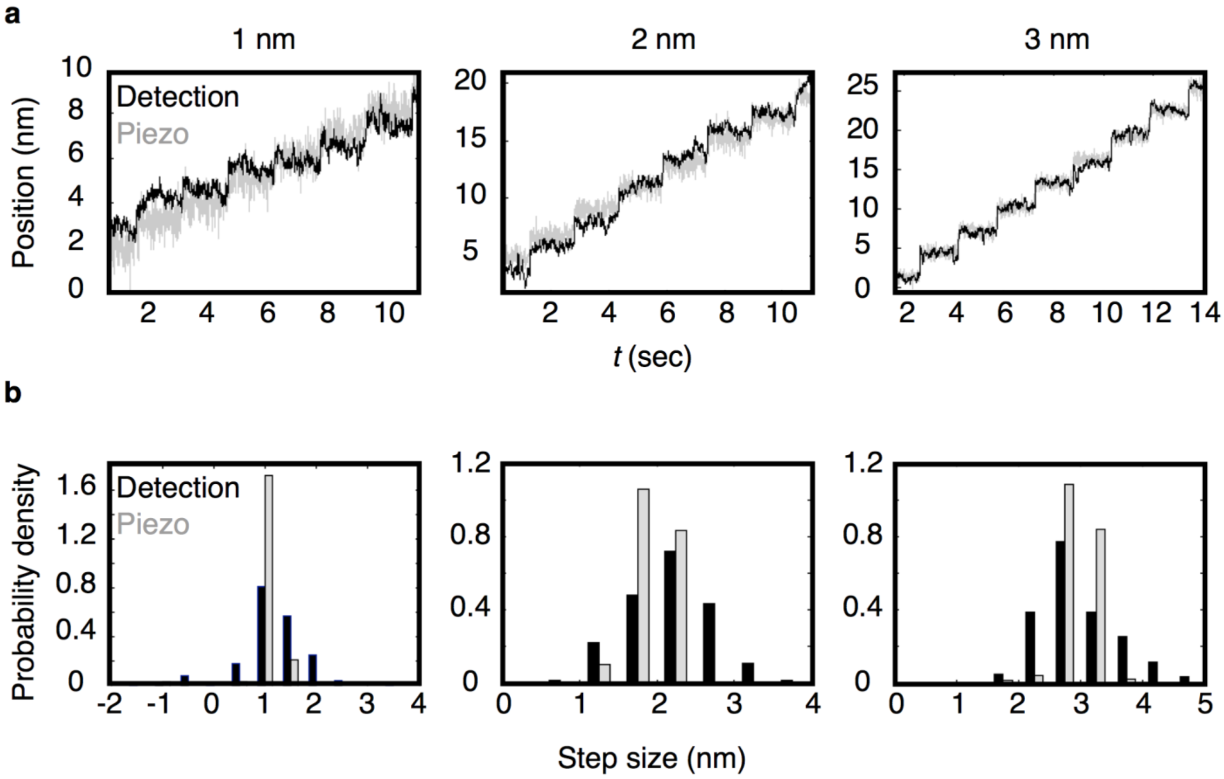

Supplementary Figure 5. Step controls – a) Sample traces of step controls showing both the movement of the piezo stage (grey) and the associated position of an immobilized bead by the detection laser (black). b) Histograms of the step sizes detected by our step-finding algorithm for both the piezo stage (grey) and detection laser data (black). This data is shown for nominal step sizes of 1 nm (N=40), 2 nm (N=75), and 3 nm (N=15) from left to right with resulting average observed step sizes (piezo / detection) of 1.15 nm/1.00 nm, 2.19 nm/1.93 nm, and 2.97 nm/2.95 nm, respectively.

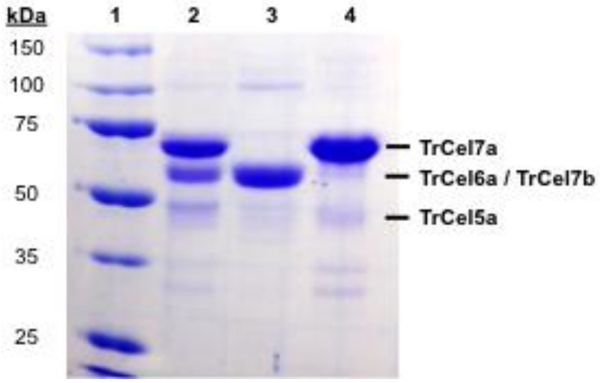

Supplementary Figure 6. wt*Tr*Cel7a purification (SDS-PAGE) – SDS-PAGE gel showing the purification TrCel7a from a commercial *Trichoderma reesei* cellulase mix. Lane 1 is a prestained protein standard whose band values are listed to the left of the image. Lane 2 is the original cellulase mixture. Lane 3 is the low salt elutant containing mostly the lighter TrCel6a and TrCel7b at 55 kDa. Lane 4 is the high salt elutant containing the desired TrCel7a at approximately 64 kDa. The light band seen at approximately 49 kDa in all lanes is due to the endoglucanase, TrCel5a.

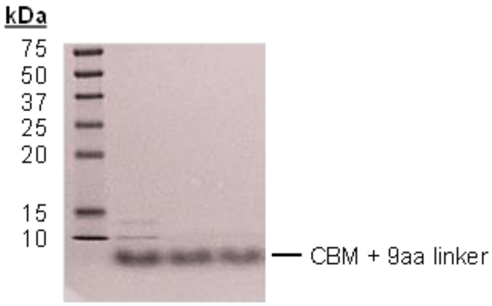

Supplementary Figure 7. Isolated CBM purification (SDS-PAGE) – A gel showing purified, isolated CBM including 9 aa of the linker domain (~6 kDa).

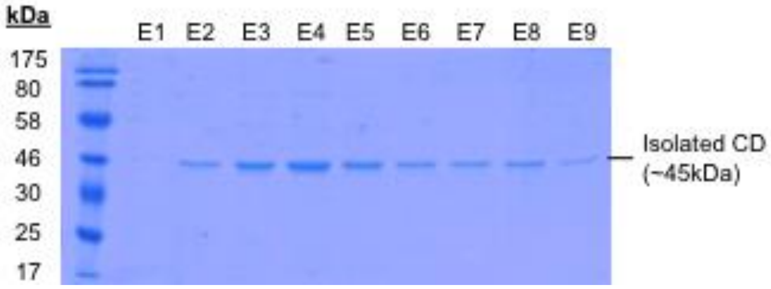

Supplementary Figure 8. Isolated CD purification (SDS-PAGE) – A gel showing purified, isolated CD domain (~45 kDa) in which each lane is a fraction of the sequential elution of CD from the HisTrap FF Ni^{2+}-NTA affinity column during purification.

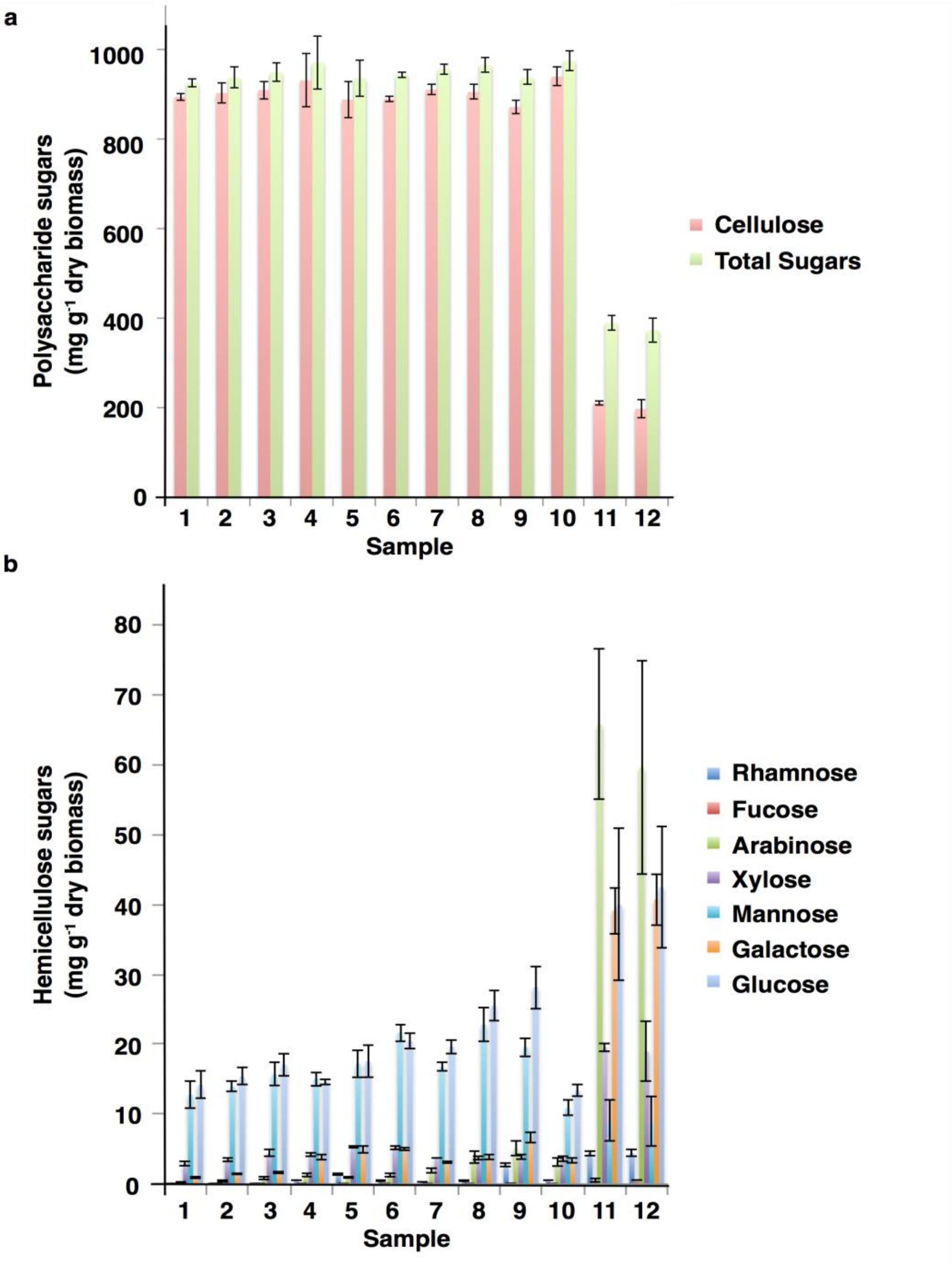

Supplementary Figure 9. *Cladophora* derived substrate carbohydrate composition – Carbohydrate compositions (dry weight basis) of twelve tested extraction conditions (% acetic acid, acetic acid solution pH, NaOH extraction solution strength and number of extractions) as outlined in **Supplementary Table 1**. Samples 11 and 12 are unextracted controls. Samples were tested for **(a)** cellulose and **(b)** hemicellulosic carbohydrate composition. All assays were carried out in triplicates with mean values reported here. Error bars indicate s.d. for reported mean values. Unextracted *Cladophora* algae biological replicates (Samples 11 and 12) were collected from two separate locations in Lake Mendota and adjoining water bodies.

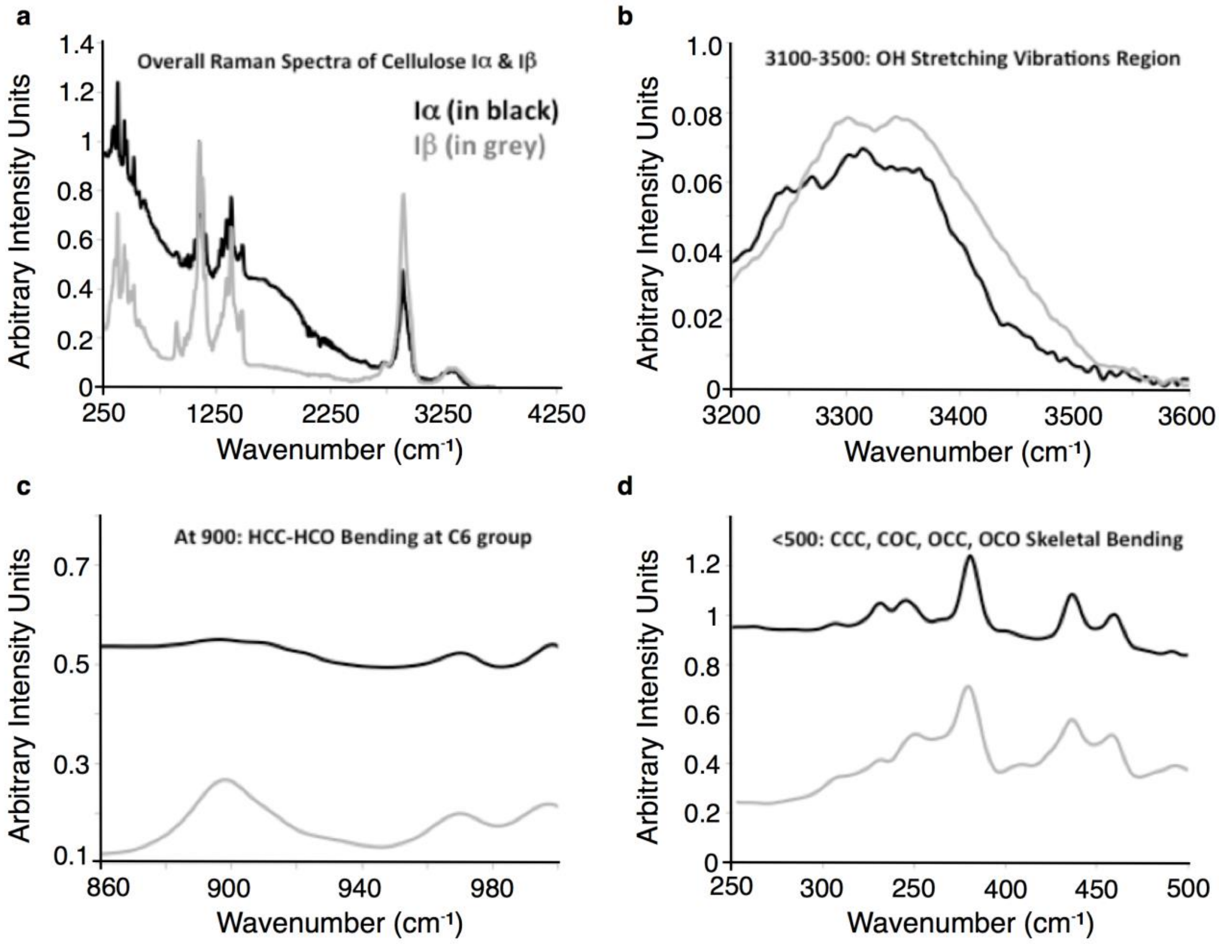

Supplementary Figure 10. Raman spectroscopy analysis of isolated *Cladophora* – *Cladophora* cellulose Iα (black lines) used in this study is compared to a cellulose Iβ control (grey lines) which is the cellulose structure present in Avicel or microcrystalline cellulose from plant-derived sources. Absence of a sharp peak at 900 cm^{-1} wavenumber confirms the highly crystalline nature of algal cellulose, unlike heterogeneous plant-derived cellulose Iβ.

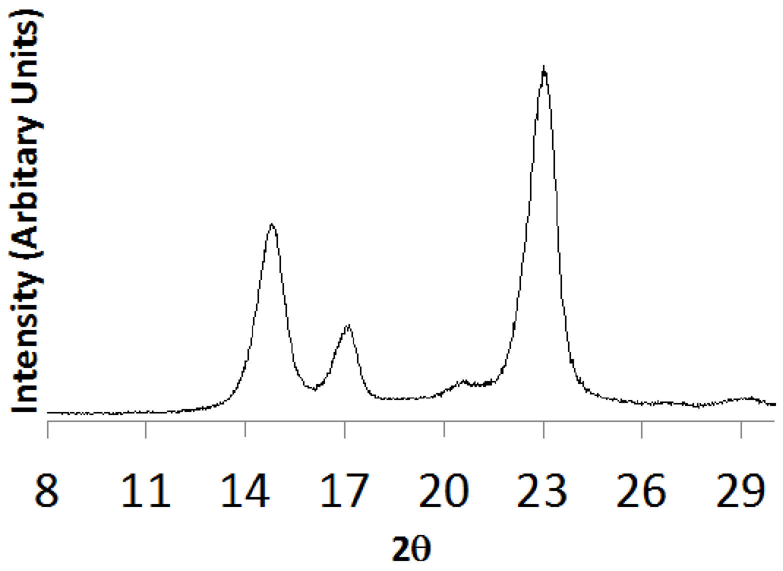

Supplementary Figure 11. X-ray diffraction spectra of *Cladophora* derived cellulose Iα – The Segal method can be used to estimate algal cellulose crystallinity index (CrI) to be ~92% ($CrI=100*(I_{23}-I_{18})/I_{18}$). Where, I_{23} and I_{18} are the XRD spectral intensities at 23 and 18 degrees two-theta values, respectively.

Supplementary Table 1. *Cladophora* cellulose extraction conditions

Sample Number	% Acetic Acid, pH	1st NaOH Extraction*	2nd NaOH Extraction*
1	1%, 2.5	1M	-
2	1%, 2.5	0.5M	-
3	1%, 2.5	0.1M	-
4	1%, 2.5	0.1M	0.5M
5	1%, 2.5	0.1M	0.1M
6	1%, 2.5	0.5M	0.5M
7	10%, 2.5	1M	-
8	10%, 4.5	1M	-
9	1%, 4.5	0.5M	-
10	1%, 4.5	0.5M	0.5M
11	Unextracted Cladophora algae biological replicate 1		
12	Unextracted Cladophora algae biological replicate 2		

*Each NaOH extraction step was carried out overnight at 60°C.

SUPPLEMENTARY NOTE 1: ENZYME ACTIVITY

The activity of expressed isolated CD and purified wt*Tr*Cel7A was specifically tested in three ways: mass spectrometry for cellobiose product, fluorescence decoration of surface bound fibers, and the filter paper assay (FPA). While bulk activity assays showed undetectable cellobiose product using the CD construct, mass spectrometry and fluorescence studies suggest activity at approximately 50 times less than that of wt*Tr*Cel7A, almost exclusively due to the absence of the CBM.

***Mass spectrometry of CD and* wt*Tr*Cel7A *activity*:** CD samples were positive for cellobiose with a sample concentration of 15.6 µM and corresponding undiluted concentration of 260 µM (0.09 mg mL^{-1}) (**Supplementary Fig. 2**). Similarly, wt*Tr*Cel7A shows a sample concentration of 50.86 µM and undiluted concentration of 15.26 mM (5.22 mg mL^{-1}). Filter paper controls show low response below the detection limit with an undiluted concentration estimated to be below 1.5 µM. Our standards reveal a detection limit of cellobiose near 1 µM while maintaining a signal to noise ratio of 10:1. These results indicate that wt*Tr*Cel7A has an apparent activity approximately 58.7 times higher than that of isolated CD. As supported in the following fiber decoration method, this difference is thought to be almost entirely due to a decrease in binding as a result of a lack of CBM and not a result of misfolded or enzymatically inactive motor.

Fluorescence decoration of fibers: Both CD and *wtTr*Cel7A enzyme specifically bound to the cellulose fibers. However, in order to observe CD binding at similar intensity to that of wt*Tr*Cel7A at 125pM, the required CD concentration was elevated 50x to 6nM (**Supplementary Fig. 3**). This result is consistent with reports by Igarashi et al[1], which state that at least 10x more CD than *Tr*Cel7A was required to observe binding using AFM. Decoration studies suggest that isolated CD is competent to bind fibers but with lower apparent activity, due to an inability to commit to cellulose binding in the absence of the CBM. This level of apparent activity reduction is consistent with that seen in mass spectrometry indicating that nearly all CD that binds is also enzymatically active. Thus, *E. coli* is capable of successful heterologous expression of active CD.

Filter paper assay (FPA): Using 1mg/mL [15µM] concentrations of wt*Tr*Cel7A and succinimidyl ester modified wt*Tr*Cel7A (wt*Tr*Cel7A*), cellobiose productions of 3.35 and 4.2 mg mL^{-1}, respectively, were seen for an incubation with filter paper substrate of 50 hours at room temperature (**Supplementary Fig. 4**). The results show that succinimidyl modification of the free lysines on the enzyme during bead preparation does not negatively affect activity. For isolated CD at 1mg mL^{-1} [22uM], the DNS assay did not reveal detectable activity. The detection limit is approximately 0.4 mg/mL cellobiose which is greater than the expected 0.07mg mL^{-1} to give a 50x reduction in apparent activity for CD.

Carbohydrate Binding Module (CBM): After determining the CBM concentration in each sample (initial, flow through, and wash), it is determined that after the flow through is removed, 48.5% of the remaining CBM was removed in the wash. Thus, over 50% of the CBM exhibit high affinity for cellulose

SUPPLEMENTARY NOTE 2: CONTROL EXPERIMENTS

Drift adjustment. To ensure the validity of our drift adjustment method, data was collected following the same translocation tracking procedure as described for wt*Tr*Cel7a except a fixed, non-functionalized 1.25 μm bead adhered to the surface was tracked rather than an enzyme functionalized bead. The relative position of the larger bead was then compared to the position of a video tracked fiducial bead (0.75 μm). Once the control bead position was adjusted for drift, it was found that 85-95% of the drift was removed from the analyzed control traces. All residual influence from drift resulted in velocities at least an order of magnitude lower than the observed average velocity of wt*Tr*Cel7a.

Step resolution. The raw signal noise is similar to the step sizes observed. Thus, we performed a number of controls using artificial motion enabled through actuating a piezo stage with surface bound beads. Here we instructed the stage to step by 1 and 2 nm intervals at an average rate consistent with our observed dwell times with one step taken every 1-3 seconds. These steps were readily re-created with our analysis programs **(Supplementary Fig. 5)**, indicating a step resolution of at least 1 nm.

SUPPLEMENTARY REFERENCE

1. Igarashi, K. *et al.* High speed atomic force microscopy visualizes processive movement of Trichoderma reesei cellobiohydrolase I on crystalline cellulose. *J. Biol. Chem.* **284,** 36186–90 (2009).
